# The Chromosome-Level Genome Assembly and Comprehensive Transcriptomes of the Razor Clam (*Sinonovacula constricta*)

**DOI:** 10.3389/fgene.2020.00664

**Published:** 2020-07-07

**Authors:** Yinghui Dong, Qifan Zeng, Jianfeng Ren, Hanhan Yao, Liyuan Lv, Lin He, Wenbin Ruan, Qinggang Xue, Zhenmin Bao, Shi Wang, Zhihua Lin

**Affiliations:** ^1^Key Laboratory of Aquatic Germplasm Resource of Zhejiang, College of Biological and Environmental Sciences, Zhejiang Wanli University, Ningbo, China; ^2^MOE Key Laboratory of Marine Genetics and Breeding, College of Marine Life Sciences, Ocean University of China, Qingdao, China; ^3^Key Laboratory of Exploration and Utilization of Aquatic Genetic Resources, Ministry of Education, College of Fisheries and Life Science, Shanghai Ocean University, Shanghai, China; ^4^Laboratory for Marine Fisheries Science and Food Production Processes, Pilot National Laboratory for Marine Science and Technology, Qingdao, China; ^5^Laboratory for Marine Biology and Biotechnology, Pilot National Laboratory for Marine Science and Technology, Qingdao, China; ^6^The Sars-Fang Centre, Ocean University of China, Qingdao, China

**Keywords:** razor clam, *Sinonovacula constricta*, genome, transcriptome, stress response

## Introduction

The Chinese razor clam *Sinonovacula constricta* (Lamarck, 1818) is a member of the *Solenidae* family of bivalve molluscs, recognizing for its straight razor-like shape and fragile shells (Morton, 1984). It is widely distributed in the intertidal zone along the west Pacific Ocean and engages in a pelago-benthic life cycle (Wang and Xu, 1997). As adaptation to a deep-burrowing lifestyle, the razor clam is characterized by smooth shells, muscular foot, and elongated siphons (Morton, 1984). Benefit from its relatively short production cycle and high productive efficiency, the razor clam has become one of most important maricultured bivalve species in Asia with over 800,000 metric tons of production in 2017 (FAO, 2019).

As living in the estuarine and intertidal region, the razor clam faces tremendous exposure to extreme environmental stresses such as drastic salinity fluctuation, highly variable temperature, high concentration of ammonia nitrogen and hydrogen sulfide (Morton, 1984). Unlike oysters, mussels and most clams with thick and sealed shells for protecting their soft bodies, the razor clam with two thin and unclosed shells has chosen a survival strategy of deep-burrowing lifestyle with high tolerance of a broad range of salinities (5–45 psu) (Morton, 1984; Peng et al., 2019). Therefore, it is an ideal model with which to investigate the adaptive mechanisms of a deep-burrowing lifestyle. Despite that increased genomic sequences (Dong et al., 2019; Ran et al., 2019) and transcriptomic data (Niu et al., 2013) have been generated, full-spectrum spatial-temporal transcriptomes are still insufficient for exploring its unique biology and adaptive evolution.

Here, we generated the high-quality chromosomal-level genome assembly and comprehensive transcriptomes of *S. constricta* and investigated the transcriptomic changes under salinity and ammonia stresses. These genomic resources will lay a prime foundation for future studies of deep-burrowing lifestyle-related adaptive evolution and genetic improvement in commercial breeding programs of razor clam.

## Data

**Figure 1:**
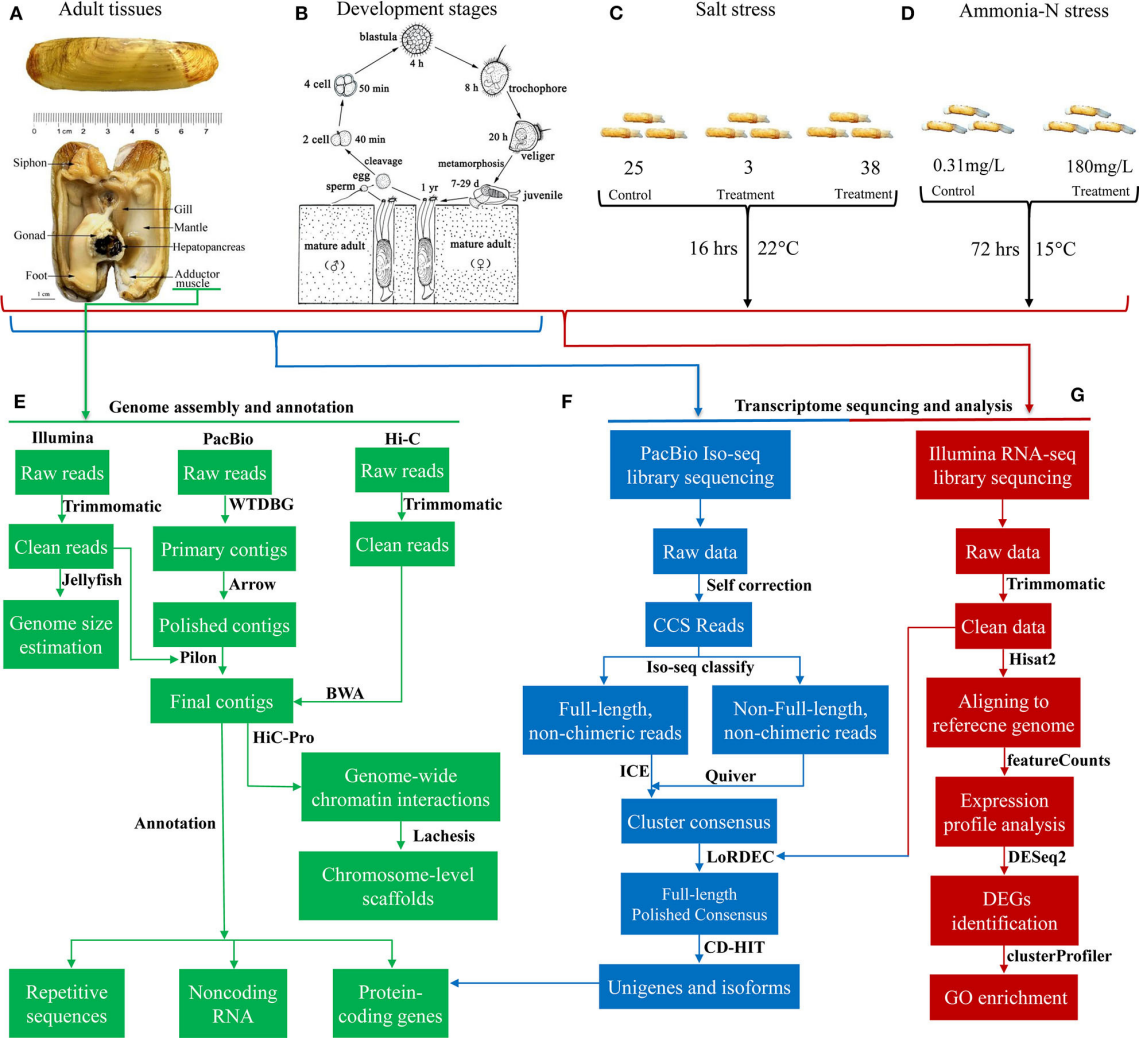
Overview of the experimental design and the data processing pipeline. Illustrations of the anatomical structure **(A)** and life cycle **(B)** of *S. constricta*. Experimental design for the salt stress **(C)** and ammonia nitrogen challenge **(D)**. Pipelines for the genome sequencing, assembly and annotation **(E)**, full-length transcriptome sequencing and analysis **(F)**, and Illumina transcriptome sequencing and analysis **(G)**.

**Figure 2:**
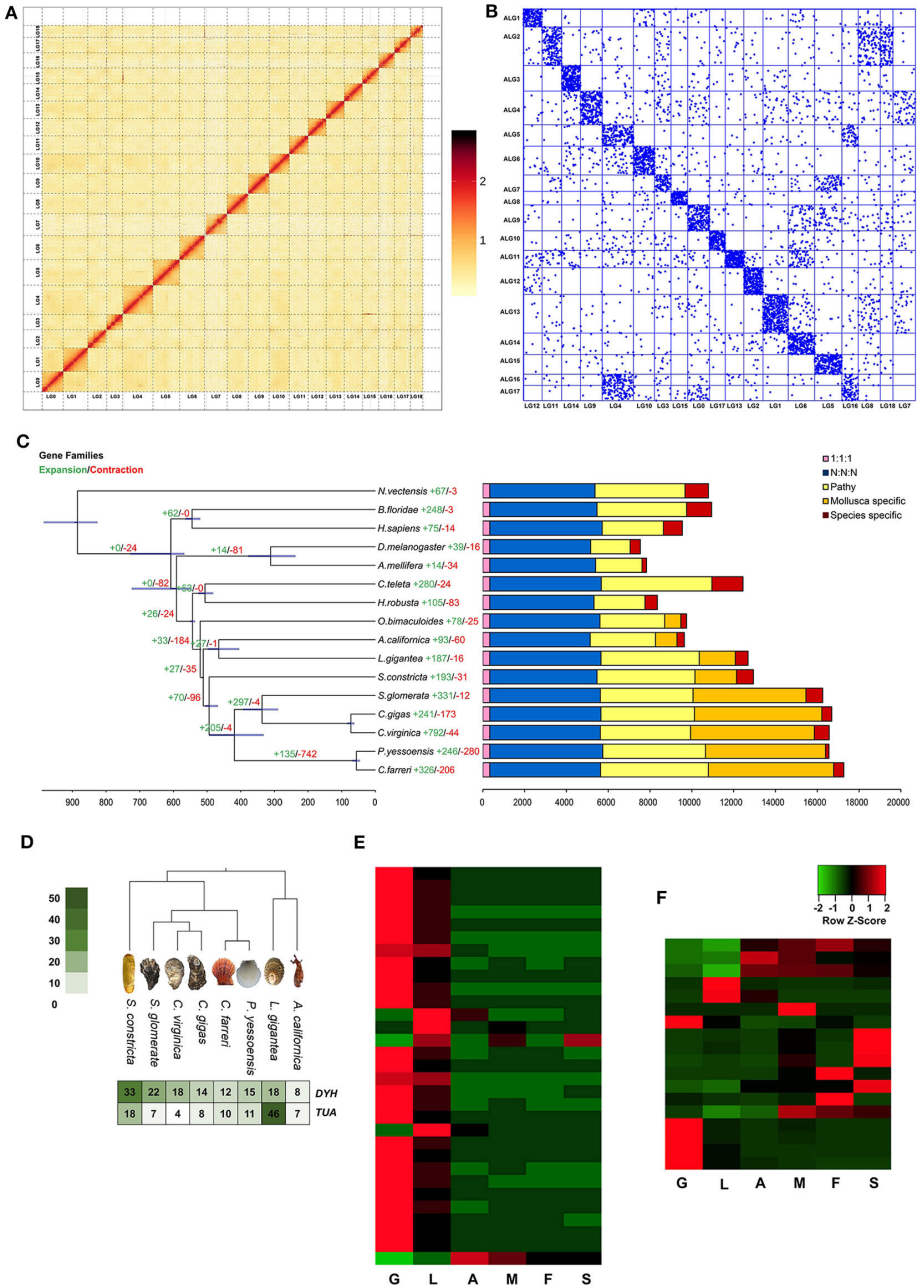
**(A)** Phylogenetic tree and number of shared orthologs among *S. constricta* and other animal species. Numbers of gene families undergoing expansion and contraction for each lineage are indicated as red and green, respectively. **(B)** The comparison of the copy numbers of dynein heavy chain (*DYH*) and alpha tubulin (*TUA*) genes in 8 molluscan species. **(C–D)** The tissue-wide expression patterns of *DYH* genes and *TUA* genes. G, gill; L, liver; A. adductor muscle; M, mantle; F, foot; S, siphon. **(E)** Hi-C interaction heat map of of *S. constricta*. **(F)** Chromosome-based macro-synteny between *S. constricta* and the 17 presumed bilaterian ALGs retrieved from Simakov et al. (2013).

The experimental design was illustrated in Figure 1. In total, 386.2 Gb clean data were used for assembling the genome of *S. constricta*, including 129.73 Gb Illumina reads, 101.79 Gb Pacbio reads, and 154.68 Gb Hi-C reads (Table 1). The genome size was estimated to be 1,244.27 Mb, with a heterozygous ratio of 1.53% and a repeat rate of 53.12%, which was consistent with the results of a previous study (Ran et al., 2019). By using the WTDBG pipeline, the resulting 1,331.97 Mb assembly was obtained with a contig N50 of 678,857 bp (Table 1). The contigs were clustered into 19 linkage groups using Lachesis (Figure 2A), which was in concordance with the karyotype analysis (Wang et al., 1998). The final assembly was 1,331.28 Mb in length and the scaffold N50 reached over 57.99 Mb. The *S. constricta* genome exhibited a remarkable level of macrosynteny to the ancestral bilaterian linkage groups with a conservation index (CI) of 0.71, indicating the high accuracy of the Hi-C clustering (Figure 2B; Simakov et al., 2013; Wang et al., 2017). The integrity of the genomic assembly was evaluated by alignment of the Illumina reads. In summary, 88.90% of the genomic sequences were covered by 93.93% of the total reads (Table S1). The Core Eukaryotic Genes Mapping Approach (CEGMA) analysis (Parra et al., 2007) and Benchmarking Universal Single-Copy Orthologs (BUSCO version 3) analysis also revealed a high-level of completeness by identifying 227 of the 248 core eukaryotic genes (91.53%) and 868 of the 978 near-universal single-copy metazoan orthologs (88.7%), respectively (Tables S2, S3).

**Table 1:** Summary statistics of genome assembly of *Sinonovacula constricta*.

**Section**	**Results**	
**A. SEQUENCES**		
	**Clean data (Gb)**	**Depth (X)**
Illumina	129.73	104.62
PacBio	101.79	82.01
Hi-C	154.68	124.74
Total	386.20	311.37
**B. ASSEMBLY**		
Number of scaffolds	7,932	
Total bases (bp)	1,332,277,427	
Contig N50 (bp)	678,857	
Scaffold N50 (bp)	57,991,182	
**C. GENES**		
Number of genes	26,270	
Complete BUSCOs	88.75%	
Number of functional annotated genes	26,140	
**D. REPETITIVE SEQUENCES**		
	**Repeat size (bp)**	**% of genome**
DNA	191,499,094	14.38
LINE	71,938,692	5.40
LTR	144,451,530	10.84
SINE	5,528,172	0.42
Tandem repeat	204,889,587	15.38
Other	157,232	0.01
Unknown	56,940,582	4.27

Genomic annotation identified 50.71% of the assembled genome as repetitive sequences. Repetitive sequences were dominated by tandem repeats (15.39%) and followed by DNA transposons (14.38%) and LTR retrotransposons (10.84%) (Table S4). Using a combination of *de novo* prediction, homologous prediction, and alignment of transcriptomic data, 26,270 protein-coding genes were annotated (Table S5). A total of 26,140 (99.5%) genes could be functionally annotated by at least one database (Table S6).

The gene set of *S. constricta* were compared to fifteen eumetazoan species, including *Homo sapiens, Branchiostoma floridae, Drosophila melanogaster, Apis mellifera, Helobdella robusta, Capitella teleta, Octopus bimaculoides, Lottia gigantea, Aplysia californica, Crassostrea gigas, Crassostrea virginica, Saccostrea glomerata, Patinopecten yessoensis, Chlamys farreri*, and *Nematostella vectensis*. The proteins from all 16 species were assigned to 39,058 families with 337 strict single-copy orthologs. Maximum likelihood phylogenetic inference of these single-copy orthologs revealed that *S. constricta* diverged from the lineage that led to oysters and scallops ~494 million years ago (Mya; Figure 2C). Among the 12,945 gene families identified in the *S. constricta* genome, 193 and 31 gene families were significantly expanded and contracted, respectively (Figure 2C). Notably, the cytoskeletal protein alpha tubulin (*TUA*) and the motor protein dynein heavy chain (*DYH*) families were rapidly expanded in the *S. constricta* genome (Figure 2D, Figures S1, S2). These families play vital roles in the microtubule architecture and bending movement of cilia (Mohri et al., 2012). The razor clam has a highly developed ciliary system for gill filtering, food-particle retention and water pumping (Morton, 1984). Adjoining cilia generate effective beats through coordinated wavelike movements. The pumping rate of the ciliary system in the gill and mantle cavity can be adjusted to generate powerful currents that facilitate the principal sorting and retaining of suspended particles in the labial palps. Effluxes can also be ejected from the pedal gape to flush away sources of irritation detected by sensory tentacles (Morton, 1984). The transcriptomic data revealed that high levels of *TUA* and *DYH* genes are expressed in the gills (Figures 2E,F), implicating that the expansion of these genes might be an adaptation to the deep-burrowing lifestyle.

Molecular adaptations of *S. constricta* to salt fluctuations were analyzed by comparing the whole transcriptomic alterations under the salinity of 3, 25, and 38 psu (Figure S3). A total of 462 upregulated and 655 downregulated genes were identified under elevated salinity. Functional analysis showed that these differentially expressed genes (DEGs) were significantly enriched in chitin metabolism, immune response, scavenger receptor activity, and ATPase activity coupled to transmembrane substance movement. When facing the decreased salinity, *S. constricta* enhanced the expression of 898 genes and suppressed 826 genes. Enrichment analysis revealed that these DEGs were mainly related to transition metal ion binding and oxidation-reduction process. Tolerance of razor clams to the ammonia nitrogen stress were investigated by transcriptome analysis (Figure S4). In the gill, 1,029 and 386 genes were upregulated and downregulated after ammonia challenge, respectively. Functional analysis indicated that DEGs were significantly enriched in the regulation of nitrogen metabolism and nitrogen transport. A smaller number of DEGs were identified in the liver, including 248 upregulated and 58 downregulated genes, respectively. GO and KEGG analysis showed that the DEGS were significant enrichments in response to stress, exopeptidase activity, and copper ion binding.

## Materials and Methods

### Genomic DNA Preparation, PacBio, and Illumina Sequencing

An individual *S. constricta* was collected from the brood stock of the genetic breeding center of Zhejiang Wanli University. Genomic DNA was extracted from the adductor muscle with phenol-chloroform as described (Green and Sambrook, 2012). High molecular-weight genomic DNA was sheared into fragments of ~30 kb using a Covaris ultrasonicator (Covaris Inc., Woburn, MA, USA). The fragments were enzymatically repaired and converted into SMRTbell™ template library following the manufacturer's instructions. Size-selection was performed to enrich the DNA fragments longer than 10 kb for sequencing on a Pacific Biosciences (PacBio) Sequel Single Molecule Real Time (SMRT) platform. A paired-end Illumina library with an insert size of 350 bp was prepared using Illumina Genomic DNA sample preparation kits and sequenced on an Illumina Xten system. Adductor muscle tissue of a razor clam from the same population was collected to construct a Hi-C library. The specimen was fixed with 1% formaldehyde and the genomic DNA was cross-linked, digested with the restriction enzyme *Mbo*I, labeled with a biotinylated residue and end repaired. The library was sequenced on an Illumina NovaSeq platform.

### Estimation of Genome Size and Sequencing Coverage

The Illumina reads were first trimmed to remove adaptors and reads with >10% ambiguous or >20% low-quality bases using the Trimmomatic package (Bolger et al., 2014). The distribution of the 17-mer frequency was estimated using the clean reads. Genome size was estimated according to the formula: genome size = k-mer number/peak depth (Varshney et al., 2011).

### *De novo* Genome Assembly and Quality Assessment

PacBio long reads were assembled using the WTDBG package (Ruan and Li, 2019). The consensus calling of preceding assembly was conducted with wtdbg-cns to reduce sequencing errors and subsequently polishing by Arrow (SMRT Link v 5.1.0). To ensure high accuracy of the genome assembly, Illumina paired-end clean reads were aligned to the assembly using BWA. Post-processing error was corrected and conflicts of the assembly were resolved via Pilon (Walker et al., 2014).

The HiC reads were truncated at the junctions and aligned to the polished genome using BWA (version 0.7.17) with default parameters. Only unique aligned reads with a mapping quality >20 were further processed. After filtering invalid interaction pairs using HiC-Pro (v.2.8.0) (Servant et al., 2015), the valid pairs were used to evaluate interaction strength among whole genome contigs. Lachesis (version 2e27abb) was used to cluster and anchor the contigs to the chromosomes using agglomerative hierarchical clustering (Burton et al., 2013).

To assess the integrity of the genome assembly, Illumina reads were mapped to the contigs using BWA (version 0.6.2). Genome completeness was also evaluated using the Core Eukaryotic Genes Mapping Approach (CEGMA) (Parra et al., 2007) and Benchmarking Universal Single-Copy Orthologs (BUSCO version 3) (Waterhouse et al., 2017).

### Repetitive Sequence Annotation

Repetitive sequences in the genome assembly were identified through *ab initio* prediction and homology-based searches. Repeat families in the *S. constricta* genome were identified *de novo* using RepeatScout (version 1.0.5) and Repeat Modeler (version 1.0.11). Full length long terminal repeat (LTR) retrotransposons were also identified using LTR-finder (version 1.0.2) (Xu and Wang, 2007) with the parameters “-E –C.” Tandem repeats were screened using Tandem Repeats Finder (TRF version 4.09) (Benson, 1999) with the parameters “match = 2, mismatching penalty = 7, indel penalty = 7, match probability = 80, indel probability = 10, minimum alignment score = 50, maximum period size = 500.” The predicted repetitive sequences along with the RepBase database (Bao et al., 2015) were used for homology-based searches using Repeatmasker (version 4.5.0) with the parameters “-a -nolow -no_is -norna -parallel 32 -small -xsmall -poly -e ncbi -pvalue 0.0001” (Tarailo-Graovac and Chen, 2009).

### Protein-Coding Gene Prediction and Annotation

Protein-coding genes were annotated based on *de novo* prediction, homology-based searches, and transcriptome-assisted methods. The protein sequences of 11 species (Table S5) were aligned to the genome assembly using TBLASTN with the parameters “-evalue 1e-5.” The gene structures were predicted using GeneWise (version 2.4.1) (Doerks et al., 2002). The Illumina RNA-seq reads of eight tissues and eight developmental stages were aligned to the genome assembly using Tophat (version 2.1.1) (Trapnell et al., 2009) and gene models were generated using Cufflinks (version 2.1.0) (Trapnell et al., 2010) with the parameter “-multi-read-correct.” The resulting GTF file and the PacBio Iso-seq transcripts were used to model gene structures with the PASA pipeline (version 2.0.2) (Haas et al., 2008). *De novo* gene prediction packages, including Augustus (version 2.5.5) (Stanke et al., 2006), glimmerHMM (version 3.01) (Majoros et al., 2004), SNAP (version 2006-07-28) (Leskovec and Sosic, 2016), Geneid (version 1.4) (Parra et al., 2000), and Genscan (version 3.1) (Burge and Karlin, 1997) were used to predict genes with repeat-masked genome sequences. All gene model evidences were integrated using EVidenceModeler (version 1.1.1) (Haas et al., 2008).

Functional annotation was performed by aligning the predicted protein sequences to public databases, including KEGG, SwissProt and NCBI-NR, using BLASTP with an E-value threshold of 1e-5. InterProScan (v.4.8) (Jones et al., 2014) was also used to identify motifs and domains obtained from searching the Pfam, InterPro, and Gene Ontology (GO) databases.

### Noncoding RNA Prediction and Annotation

The noncoding RNA genes, including miRNA, rRNA, snRNA, and tRNA, were annotated in the *S. constricta* genome. Transfer RNA were predicated by tRNAscan-SE 2.0 (Lowe and Chan, 2016) with eukaryote parameters. The miRNA and snRNA were screened using INFERNAL 1.1.2 against the Rfam database (version 14.1) (Kalvari et al., 2018) with default parameters (Table S7).

### Gene Family Identification and Phylogenetic Analysis

Fifteen Eumetazoan species were selected for gene family analysis. All data were downloaded from either NCBI or Ensembl. The longest protein sequence was selected to represent a gene with multiple alternative splicing isoforms. Gene family clusters from all 16 species were first assigned using OrthoMCL (version 2.0.9) (Li et al., 2003) with an inflation value of 1.5. Gene family expansion and contraction under a maximum likelihood framework were analyzed using CAFE (version 3) (De Bie et al., 2006).

Phylogenetic inferences of the 16 species were conducted using 337 single-copy orthologs. Multiple sequences were aligned for the protein sequences of each ortholog gene using MUSCLE (version 3.8.31) (Edgar, 2004). The alignments of all the orthologs were then concatenated into a super alignment matrix. RAxML (version 8.2.12) (Stamatakis, 2014) was used to infer the alignment matrix via a maximum likelihood method with the substitution model PROTGAMMAAUTO. Node support was provided by bootstrapping with 100 replicates. The time when species diverged was estimated using MCMCTree in the PAML package (version 4.7a) (Yang, 1997) with the parameters of “burn-in = 1,000, sample-number = 1,000,000, sample-frequency = 2.”

### Transcriptomic Sequencing and Analysis

Embryos (eggs, four cells, blastulae, gastrulae), larvae (trochophore, D-shaped, umbo larvae and juvenile) and adults of *S. constricta* were collected for RNA sequencing. Eight tissues/organs (gill, liver, foot, mantle, adductor muscle, siphon, ovary, and testis) were dissected from three individuals. All the samples were flash frozen in liquid nitrogen and stored at −80°C until use. Full-length RNA was sequenced using mixed RNA from samples of the eight development stages and eight adult tissues. Three libraries with different insert lengths (e.g., 1–2k, 2–3k, and 3–6k) were constructed and sequenced on a PacBio Sequel platform. The full-length RNA transcriptome was analyzed using the SMRT Link software (v4.0.0). After redundant sequence clustering using the ICE algorithm, consensus sequence building using the DAGCon algorithm and polishing with Quiver. The transcripts were then polished and corrected using Illumina reads with LoRDEC (Salmela and Rivals, 2014). Finally, the corrected transcripts were further clustered with CD-HIT (version 4.6) (Li and Godzik, 2006).

To identify genes and pathways involved in the tolerance to salt fluctuations and ammonia. *S. constricta* were subjected to salt stress for 16 h under 22°C at low-salinity (3 psu), high-salinity (38 psu), and normal-salinity (25 psu). Three replicate tanks were set for each group with 10 individuals for each replicate. Gills were dissected from three individuals of each replicate and stored at −80°C. For the ammonia challenge, two groups of *S. constricta* were subjected to total ammonia nitrogen (TAN) at the concentration of 180 and 0.31 mg/L for 72 h (15°C, 23%0 sea water and pH 8.17). Three replicate tanks were set for each group with 80 individuals per tank. Gill and liver samples were dissected from three individuals of each replicate and stored at −80°C. Total mRNA were extracted using TRIzol (Omega Bio-Tek Inc., Norcross, GA, USA). RNA libraries were constructed and sequenced on an Illumina X Ten system. Raw reads were then filtered using Trimmomatic (Bolger et al., 2014) (Table S8). The clean reads were aligned to the indexed reference genome using Hisat2 version 2.0.5 (Kim et al., 2015). Read numbers mapped to each gene were counted using featureCounts version 1.5.0 (Liao et al., 2014). Differentially expressed genes (DEG) between different experiment groups were identified using the DESeq2 R package version 1.16.1 with an adjusted *P* < 0.05 (Love et al., 2014). Gene Ontology (GO) enrichment of DEG was analyzed using the cluster Profiler 3.4.4 R package with corrected *P* < 0.05 being taken as significantly enriched GO terms (Yu et al., 2012).

## Data Availability Statement

The datasets generated for this study can be found in the NCBI under accession numbers WSYO00000000.1. The raw data of the PacBio, Illumina, and Hi-C sequencing for genome assembly are deposited at the NCBI Sequence Read Archive (SRA) with the accession numbers SRR10605359–SRR10605381, SRR10583055, and SRR10586373. The raw data of transcriptome sequencing are available at the NCBI SRA with the accession numbers SRR10753889–SRR10753895, SRR2162883, SRR2162887, SRR2162892, SRR2162895, SRR2162898, SRR2162902, SRR9937008–SRR9937013, SRR10097413–SRR10097424, SRR9959746–SRR9959754, and SRR9943679–SRR9943690.

## Ethics Statement

All experimental procedures were approved by the Institutional Animal Care and Use Committee (IACUC) of Zhejiang Wanli University, China.

## Author Contributions

YD, ZL, and ZB conceived the project. HY, WR, and LH conducted the environmental stress and collected the samples. QZ, SW, JR, and LL performed the genome assembly, annotation, transcriptome analysis and other bioinformatics analysis. YD, JR, QZ, SW, and QX wrote and revised the manuscript. All authors read and approved the final manuscript.

## Conflict of Interest

The authors declare that the research was conducted in the absence of any commercial or financial relationships that could be construed as a potential conflict of interest.
