# Supplementary material for: The Chromosome-Level Genome Assembly and Comprehensive Transcriptomes of the Razor Clam (Sinonovacula constricta)
Source: Front Genet. 2020 Jul 7;11:664. doi: 10.3389/fgene.2020.00664 (PMC7358530; doi:10.3389/fgene.2020.00664)
Supplement: Supplementary file 1 [file Data_Sheet_1.DOCX]

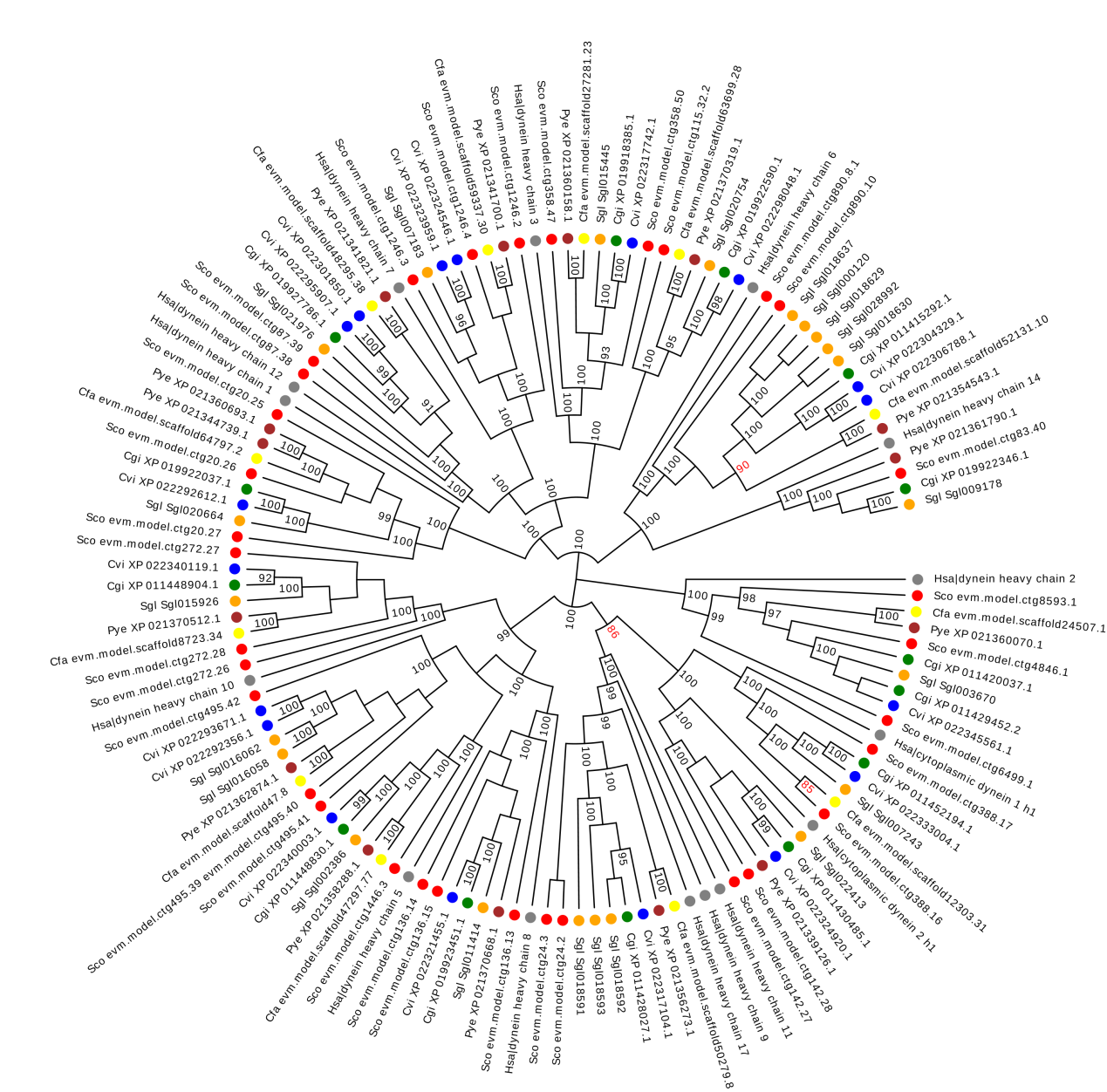


**Fig. S1** Maximum-likelihood tree of dynein heavy chain genes from *Sinonovacula constricta* and six other species. Numbers at each node indicate the bootstrap supporting values. The abbreviations for each species are as follows: Sco, *S. constricta*; Sgl*, Saccostrea glomerata*; Hsa*, Homo sapiens*; Cfa*, Chlamys farreri*; Pye*, Patinopecten yessoensis*; Cgi*,* *Crassostrea gigas*; Cvi*,* *Crassostrea virginica.*


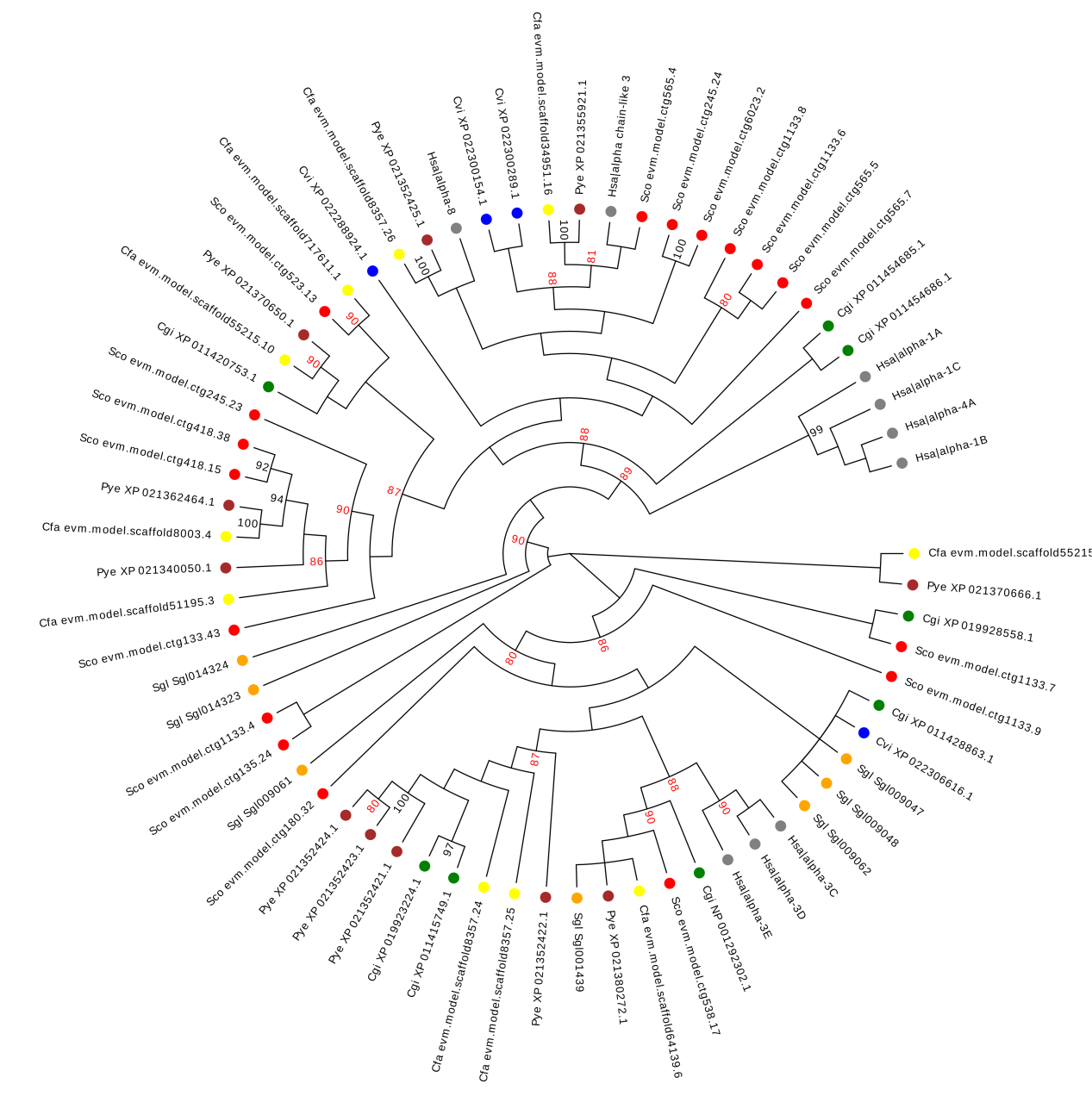


**Fig. S2** Maximum-likelihood tree of Alpha tubulin genes from *Sinonovacula constricta* and six other species. Numbers at each node indicate the bootstrap supporting values. The abbreviations for each species are as follows: Sco, *S. constricta*; Sgl*, Saccostrea glomerata*; Hsa*, Homo sapiens*; Cfa*, Chlamys farreri*; Pye*, Patinopecten yessoensis*; Cgi*,* *Crassostrea gigas*; Cvi*,* *Crassostrea virginica.*

**
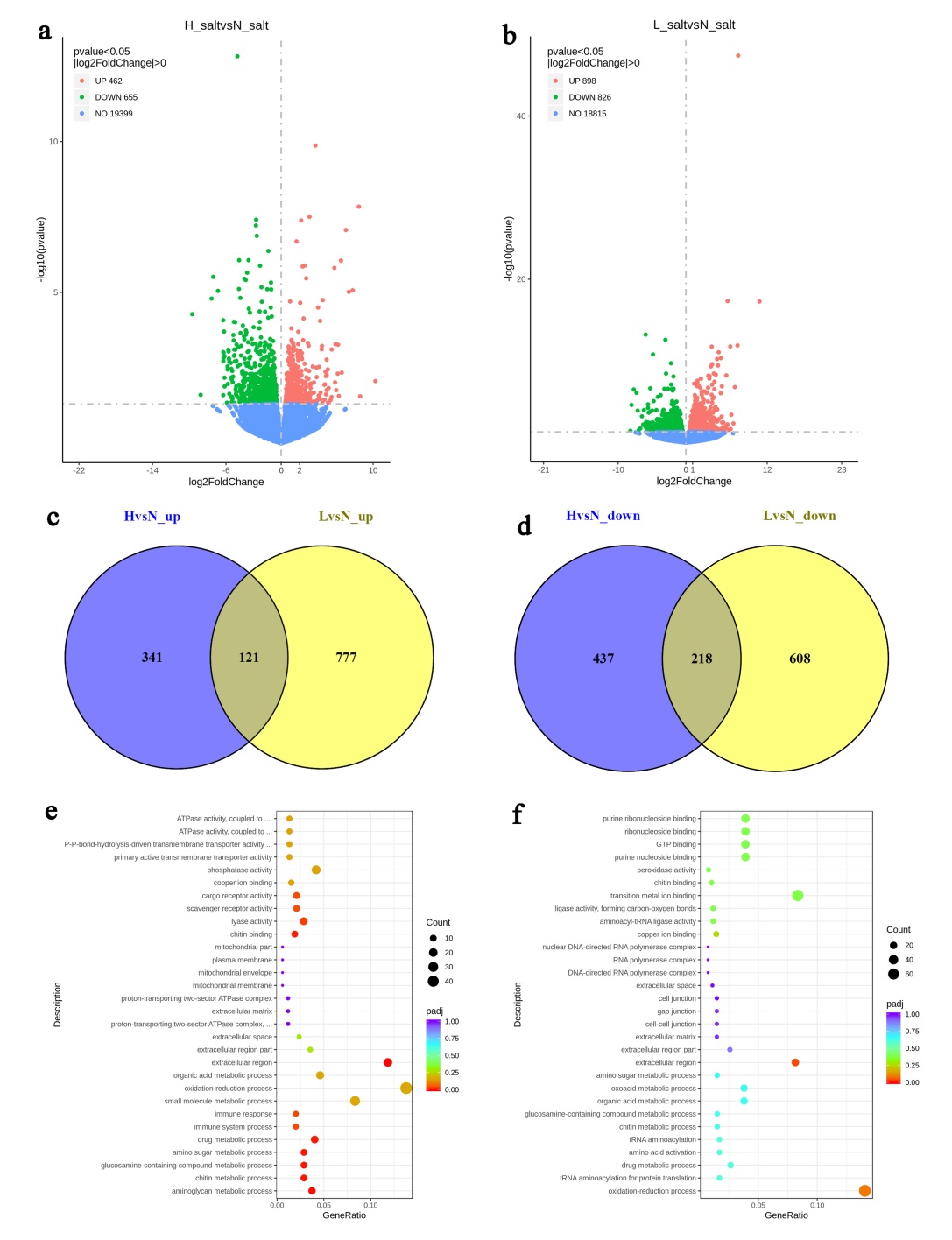
**

**Fig. S3** Transcriptome analysis of differentially expressed genes (DEGs) between groups under salt stress. (a) Volcano maps of DEGs between high-salinity group and normal-salinity group. (b) Volcano maps of DEGs between low-salinity group and normal-salinity group. (c) Venn diagram of shared up-regulated genes between the high-salinity group vs. normal-salinity group and the low-salinity group vs. normal-salinity. (d) Venn diagram of shared down-regulated genes between high-salinity group vs. normal-salinity group and low-salinity group vs. normal-salinity. (e) GO enrichment of DEGs between high-salinity group and normal-salinity group. (f) GO enrichment of DEGs between low-salinity group and normal-salinity group.


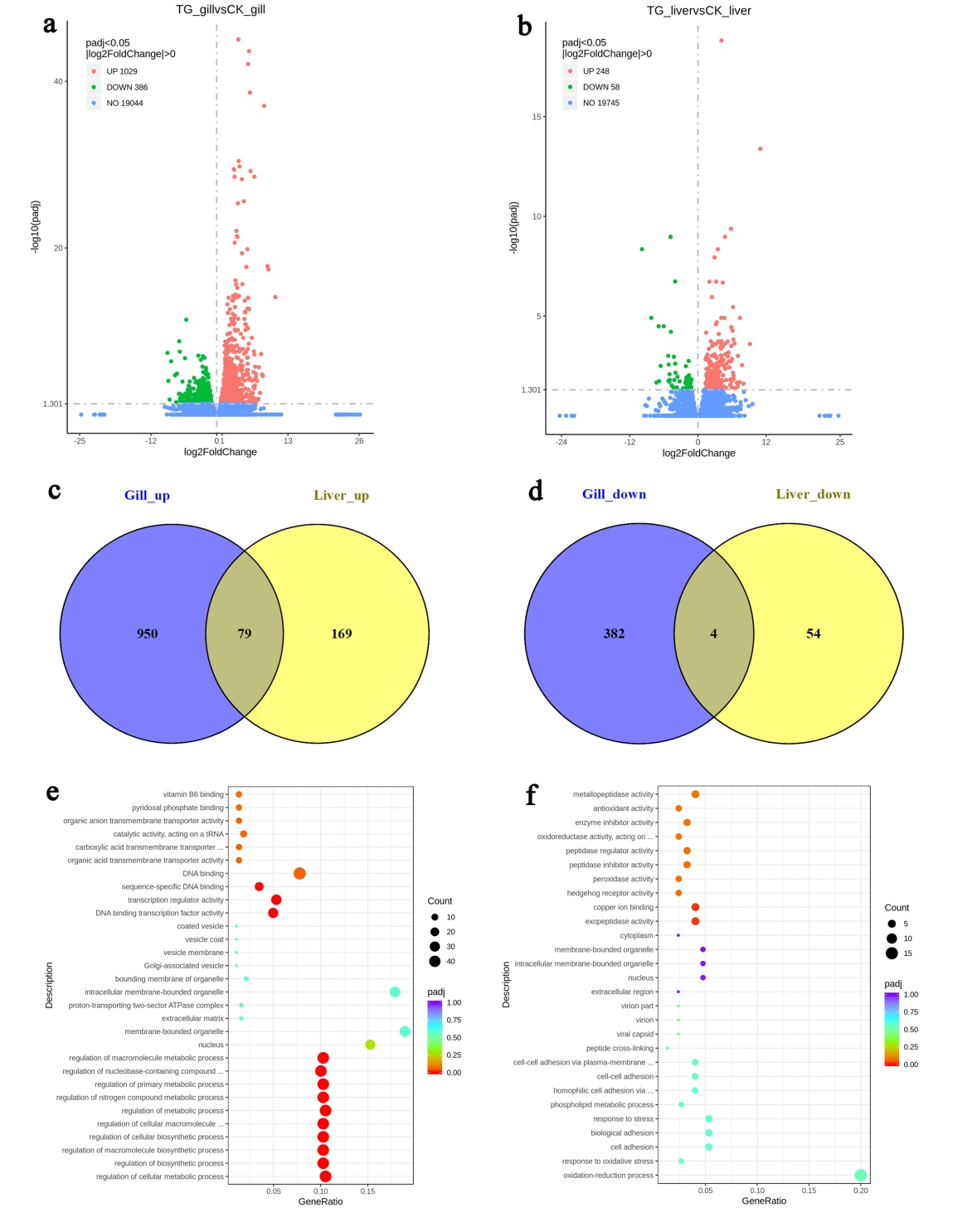


**Fig. S4** Transcriptome analysis of DEGs between groups under ammonia nitrogen stress. (a) Volcano maps of DEGs between the treat group and control group of gill tissues. (b) Volcano maps of DEGs between the treat group and control group of liver tissues. (c) Venn diagram of shared up-regulated genes between the treat group vs. control group of gills and the treat group vs. control group of livers. (d) Venn diagram of shared down-regulated genes between the treat group vs. control group of gills and the treat group vs. control group of livers. (e) GO enrichment of DEGs between the treat group and control group of gills. (f) GO enrichment of DEGs between the treat group and control group of livers**.**
