# Supplementary material for: The Chromosome-Level Genome Assembly and Comprehensive Transcriptomes of the Razor Clam (Sinonovacula constricta)
Source: Front Genet. 2020 Jul 7;11:664. doi: 10.3389/fgene.2020.00664 (PMC7358530; doi:10.3389/fgene.2020.00664)
Supplement: Supplementary file 2 [file Data_Sheet_2.DOCX]

**Table S1. Statistics of Illumina short reads coverage**

| Genome assembly | Parameter |
| --- | --- |
| Average sequencing depth | 87.11 |
| Mapping rate (%) | 93.93 |
| Coverage (%) | 88.90 |
| Coverage at least 4X (%) | 86.67 |
| Coverage at least 10X (%) | 85.18 |
| Coverage at least 20X (%) | 83.65 |

**Table S2. Summary genomic completeness by CEGMA.**

| Number of core eukaryotic genes | Complete | | | Complete and Partial | | |
| --- | --- | --- | --- | --- | --- | --- |
|  | Number | | Percentage (%) | Number | | Percentage |
| 248 | 198 | 79.84 | | 227 | 91.53 | |

**Table S3. Summary genomic completeness by BUSCO.**

| Number of core metazoan genes | Complete | | Fragmented | Missing |
| --- | --- | --- | --- | --- |
|  | single copy | duplicated |  |  |
| 978 | 832 | 36 | 40 | 70 |

**Table S4. Statistics of the repetitive sequences**

| Annotation Method | Repeat Size  (bp) | % of  Genome |
| --- | --- | --- |
| Trf | 347,649,222 | 26.10 |
| Repeatmasker | 486,560,380 | 36.53 |
| Proteinmask | 43,792,830 | 3.29 |
| Total | 675,404,889 | 50.71 |

**Table S5. Summary of the gene prediction results**

| Method | Software | Species | Gene number |
| --- | --- | --- | --- |
| *Ab initio* | Augustus | - | 72,557 |
|  | GlimmerHMM | - | 231,927 |
|  | Genscan | - | 53,981 |
|  | GeneID | - | 35,803 |
|  | SNAP | - | 127,520 |
| Homology-based | GeMoMa | *Branchiostoma floridae* | 39,275 |
|  |  | *Caenorhabditis elegans* | 5,479 |
|  |  | *Crassostrea gigas* | 27,530 |
|  |  | *Ciona intestinalis* | 10,680 |
|  |  | *Drosophila melanogaster* | 5,741 |
|  |  | *Helobdella robusta* | 27,467 |
|  |  | *Homo sapiens* | 12,495 |
|  |  | *Lottia gigantea* | 61,176 |
|  |  | *Octopus bimaculoides* | 18,868 |
|  |  | *Patinopecten yessoensis* | 41,949 |
|  |  | *Strongylocentrotus purpuratus* | 20,929 |
| RNA-seq | Full-length seq | - | 75,225 |
|  | Cufflinks |  | 69,612 |
|  | PASA | - | 30,235 |
| Integration | EVM | - | 40,123 |
|  | PASA-update |  | 26,270 |

**Table S6. Statistics of gene annotation to different databases.**

| Annotation database | Number of annotated genes | Percentage |
| --- | --- | --- |
| NR | 23,844 | 90.88 |
| Swiss-Prot | 18,131 | 69.1 |
| KEGG | 18,928 | 72.14 |
| InterProScan | 25,475 | 97.1 |
| Pfam | 15,391 | 58.66 |
| GO | 22,956 | 87.49 |
| Total | 26,140 | 99.50 |

**Table S7. Summary of the non-coding RNA annotation**

| Type | Copy | Average length (bp) | Total length (bp) |
| --- | --- | --- | --- |
| miRNA | 968 | 102.75 | 99,462 |
| tRNA | 3354 | 74.58 | 250,141 |
| 18S rRNA | 516 | 321 | 165,636 |
| 28S rRNA | 65 | 115.95 | 7,537 |
| 5S rRNA | 241 | 90.95 | 21,919 |
| CD-box snRNA | 67 | 88.73 | 5,945 |
| HACA-box snRNA | 37 | 183.56 | 6,792 |
| Splicing snRNA | 193 | 147.58 | 28,483 |

**Table S8. Summary of Illumina short-read transcriptome sequencing.**

| Sample type | Sample name | Raw reads | Clean reads | Clean bases (Gb) | Total mapped | Mapping rate |
| --- | --- | --- | --- | --- | --- | --- |
| Development stages | Egg | 43,935,046 | 42,881,284 | 6.43 | 24,187,623 | 56.41% |
|  | Four cells | 43,085,794 | 42,600,768 | 6.39 | 20,200,370 | 47.42% |
|  | Blastulae | 47,526,530 | 46,718,170 | 7.01 | 25,396,271 | 54.36% |
|  | Gastrulae | 43,935,046 | 42,881,284 | 6.43 | 24,187,626 | 56.41% |
|  | Trochophore | 43,935,046 | 42,881,284 | 6.43 | 24,187,619 | 56.41% |
|  | D-stage larvae | 36,806,136 | 36,163,274 | 5.42 | 6,791,195 | 18.78% |
|  | Umbo larvae | 43,492,564 | 43,044,208 | 6.46 | 24,011,371 | 55.78% |
|  | Juvenile | 44,296,144 | 43,739,942 | 6.56 | 25,315,001 | 57.88% |
| Adult tissues | Gill | 58,314,622 | 52,571,902 | 5.31 | 42,408,674 | 80.67% |
|  | Foot | 57,279,662 | 51,774,716 | 5.23 | 43,445,527 | 83.91% |
|  | Adductor muscle | 50,833,380 | 45,661,534 | 4.61 | 40,224,430 | 88.09% |
|  | Digestive gland | 50,796,292 | 45,624,660 | 4.61 | 36,325,819 | 79.62% |
|  | Mantle | 54,234,040 | 48,455,080 | 4.89 | 38,728,162 | 79.93% |
|  | Siphon | 54,593,774 | 49,368,016 | 4.99 | 41,079,077 | 83.21% |
|  | Ovary01 | 34,903,986 | 34,468,792 | 4.33 | 25,108,177 | 72.84% |
|  | Ovary02 | 36,188,356 | 35,741,542 | 4.50 | 25,149,497 | 70.36% |
|  | Ovary03 | 36,718,496 | 36,258,132 | 4.56 | 25,481,341 | 70.28% |
|  | Testis01 | 32,433,246 | 32,024,558 | 4.03 | 21,917,877 | 68.44% |
|  | Testis02 | 28,685,592 | 28,336,062 | 3.56 | 20,012,672 | 70.63% |
|  | Testis03 | 28,626,282 | 28,264,944 | 3.56 | 19,934,211 | 70.53% |
| Salt stress | N_salt01 | 40,034,734 | 39,581,252 | 5.83 | 27,870,270 | 70.41% |
|  | N_salt02 | 44,533,952 | 44,056,944 | 6.48 | 29,653,739 | 67.31% |
|  | N_salt03 | 44,718,238 | 44,227,004 | 6.50 | 30,710,518 | 69.44% |
|  | H_salt01 | 40,847,898 | 40,374,444 | 5.92 | 27,098,825 | 67.12% |
|  | H_salt02 | 39,929,882 | 39,491,552 | 5.80 | 27,423,141 | 69.44% |
|  | H_salt03 | 39,319,852 | 38,959,456 | 5.71 | 26,790,941 | 68.77% |
|  | L_salt01 | 40,036,290 | 39,619,590 | 5.82 | 27,108,102 | 68.42% |
|  | L_salt02 | 43,389,812 | 42,822,802 | 6.31 | 29,175,853 | 68.13% |
|  | L_salt03 | 49,465,626 | 48,917,228 | 7.22 | 33,534,046 | 68.55% |
| Ammonia nitrogen stress | CK_gill01 | 46,352,328 | 44,879,776 | 6.72 | 32,665,206 | 72.78% |
|  | CK_gill02 | 41,173,924 | 39,806,810 | 5.96 | 28,375,170 | 71.28% |
|  | CK_gill03 | 53,350,356 | 52,349,692 | 7.8 | 38,794,212 | 74.11% |
|  | CK_liver01 | 48,457,610 | 47,321,004 | 7.07 | 34,385,645 | 72.66% |
|  | CK_liver02 | 51,489,774 | 50,275,468 | 7.52 | 35,885,565 | 71.38% |
|  | CK_liver03 | 47,243,064 | 46,163,320 | 6.91 | 32,758,689 | 70.96% |
|  | TG_gill01 | 49,728,640 | 48,603,280 | 7.27 | 34,880,190 | 71.77% |
|  | TG_gill02 | 45,918,180 | 44,886,696 | 6.72 | 33,194,601 | 73.95% |
|  | TG_gill03 | 55,087,602 | 53,844,418 | 8.06 | 38,983,895 | 72.40% |
|  | TG_liver01 | 48,314,008 | 47,190,368 | 7.04 | 33,925,631 | 71.89% |
|  | TG_liver02 | 48,330,144 | 47,191,178 | 7.06 | 33,663,638 | 71.33% |
|  | TG_liver03 | 48,063,138 | 46,949,294 | 7.03 | 33,861,230 | 72.12% |
